# Oral Immediate-Release Nifedipine Versus Intravenous Hydralazine for Controlling Severe Hypertension in Pregnancy: A Double-Blind Randomised Controlled Trial

**DOI:** 10.55640/ijmsdh-12-01-10

**Published:** 2026-01-23

**Authors:** Ayodele Obianuju Okwuosa, George Uchenna Eleje, Okechukwu Christian Ikpeze, Emmanuel Onyebuchi Ugwu, Boniface Chukwuneme Okpala, Golibe Christian Ikpeze, Ifeanyi Ogochukwu Okonkwo, Hillary Ikechukwu Obiagwu, Odigonma Zinobia Ikpeze, Ifeanyichukwu Jude Ofor, Emeka Philip Igbodike, Joseph Odirichukwu Ugboaja, Kingsley Emeka Ekwuazi, Chukwuemeka Chukwubuikem Okoro, Chigozie Geoffrey Okafor, Onyecherelam Monday Ogelle, Chukwunonso Isaiah Enechukwu, Lazarus Ugochukwu Okafor, Osita Samuel Umeononihu, Zebulon Chiawolamoke Okechukwu, Chukwudubem Chinagorom Onyejiaka, Chijioke Ogomegbunam Ezeigwe, Adanna Vivian Egwim, Chukwunwendu Aloysius Okeke, Johnbosco Emmanuel Mamah, Joseph Ifeanyichukwu Ikechebelu, Ahizechukwu Chigoziem Eke

**Affiliations:** Department of Obstetrics and Gynaecology, Nnamdi Azikiwe University Teaching Hospital Nnewi, Anambra State, Nigeria; Department of Obstetrics and Gynaecology, Nnamdi Azikiwe University Teaching Hospital Nnewi, Anambra State, Nigeria. / Effective Care Research Unit, Department of Obstetrics and Gynaecology, Nnamdi Azikiwe University, Awka, Nigeria; Department of Obstetrics and Gynaecology, Nnamdi Azikiwe University Teaching Hospital Nnewi, Anambra State, Nigeria; Department of Obstetrics and Gynaecology, College of Medicine, University of Nigeria Ituku-Ozalla, Enugu, Nigeria; Department of Obstetrics and Gynaecology, Nnamdi Azikiwe University Teaching Hospital Nnewi, Anambra State, Nigeria. / Effective Care Research Unit, Department of Obstetrics and Gynaecology, Nnamdi Azikiwe University, Awka, Nigeria; Department of Obstetrics and Gynaecology, Nnamdi Azikiwe University Teaching Hospital Nnewi, Anambra State, Nigeria; Department of Obstetrics and Gynaecology, Nnamdi Azikiwe University Teaching Hospital Nnewi, Anambra State, Nigeria; Department of Obstetrics and Gynaecology, Nnamdi Azikiwe University Teaching Hospital Nnewi, Anambra State, Nigeria; Department of Obstetrics and Gynaecology, Nnamdi Azikiwe University Teaching Hospital Nnewi, Anambra State, Nigeria; Department of Obstetrics and Gynaecology, ESUT Teaching Hospital, Enugu, Nigeria; Department of Obstetrics and Gynaecology, Kelina Hospital, Victoria Island, Lagos, Nigeria; Department of Obstetrics and Gynaecology, Nnamdi Azikiwe University Teaching Hospital Nnewi, Anambra State, Nigeria; Department of Obstetrics and Gynaecology, College of Medicine, University of Nigeria Ituku-Ozalla, Enugu, Nigeria; Department of Obstetrics and Gynaecology, Nnamdi Azikiwe University Teaching Hospital Nnewi, Anambra State, Nigeria; Department of Obstetrics and Gynaecology, Nnamdi Azikiwe University Teaching Hospital Nnewi, Anambra State, Nigeria; Department of Obstetrics and Gynaecology, Nnamdi Azikiwe University Teaching Hospital Nnewi, Anambra State, Nigeria; Department of Obstetrics and Gynaecology, Nnamdi Azikiwe University Teaching Hospital Nnewi, Anambra State, Nigeria; Department of Obstetrics and Gynaecology, Nnamdi Azikiwe University Teaching Hospital Nnewi, Anambra State, Nigeria; Department of Obstetrics and Gynaecology, Nnamdi Azikiwe University Teaching Hospital Nnewi, Anambra State, Nigeria; Department of Obstetrics and Gynaecology, Nnamdi Azikiwe University Teaching Hospital Nnewi, Anambra State, Nigeria; Department of Obstetrics and Gynaecology, Nnamdi Azikiwe University Teaching Hospital Nnewi, Anambra State, Nigeria; Department of Obstetrics and Gynaecology, Nnamdi Azikiwe University Teaching Hospital Nnewi, Anambra State, Nigeria; Department of Obstetrics and Gynaecology, Nnamdi Azikiwe University Teaching Hospital Nnewi, Anambra State, Nigeria; Department of Obstetrics and Gynaecology, Nnamdi Azikiwe University Teaching Hospital Nnewi, Anambra State, Nigeria; Department of Obstetrics and Gynaecology, Alex Ekwueme Federal University Teaching Hospital, Abakaliki, Nigeria; Department of Obstetrics and Gynaecology, Nnamdi Azikiwe University Teaching Hospital Nnewi, Anambra State, Nigeria. Effective Care Research Unit, Department of Obstetrics and Gynaecology, Nnamdi Azikiwe University, Awka, Nigeria; Division of Maternal Fetal Medicine, Department of Gynecology and Obstetrics, Johns Hopkins University School of Medicine, Baltimore, Maryland, USA

**Keywords:** Severe hypertension in pregnancy, Oral immediate-release nifedipine, Intravenous hydralazine, Randomised controlled trial, Hypertensive disorders of pregnancy, Maternal and fetal outcomes, Acute blood pressure control, Low- and middle-income countries

## Abstract

**Background:**

A significant gap exists in understanding the efficacy and safety of oral immediate-release nifedipine and intravenous hydralazine in the control of severe hypertension in pregnancy within the context of randomised control trials.

**Objectives:**

To compare the efficacy and safety of oral immediate-release nifedipine and intravenous hydralazine for controlling severe hypertension in pregnancy.

**Methods:**

Randomised, double-blind, 2-arm, single center non-inferiority trial in pregnant women with confirmed severe hypertension in pregnancy who were randomised in a 1:1 ratio to receive oral immediate-release Nifedipine (n=35) or intravenous hydralazine (n=35) was done from 20^th^ June 2019 to 20^th^ December, 2019. The primary outcome was the mean time required to achieve target blood pressure. Secondary outcomes included the mean number of anti-hypertensive doses needed, proportions requiring crossover or rescue therapy, frequency of maternal side effects, fetal heart rate abnormalities, maternal complications, neonatal birth asphyxia, and mode of delivery.

**Results:**

The baseline socio-demographic parameters were similar between the two groups. The mean durations taken to reach the target blood pressure for patients that received oral immediate-release nifedipine versus intravenous hydralazine were 48.29min±31.95 and 41.20min±26.98 respectively (P=0.320). The mean dose used before target blood pressure was reached, the proportion of participants that crossed over to another treatment allocation, and the proportion that required a rescue antihypertensive (labetalol) to achieve the target blood pressure were similar between the two groups (P >0.05). None of the participants that received oral immediate-release nifedipine had maternal or neonatal side effect while one participant (2.9%) that received intravenous hydralazine had nausea and vomiting

**Conclusion:**

Oral immediate-release nifedipine is as effective as intravenous hydralazine in controlling severe hypertension in pregnancy. Both have remarkable materno-fetal safety profile and its non-invasive nature makes it an appealing modality especially in resource poor countries. More robust studies are encouraged to increase the evidence for its use as first line antihypertensives, especially in low and middle-income countries.

**Trial Registration:**

Pan African Clinical Trial registry, PACTR201906662822573, registration date: 19^th^ June, 2019.

## Introduction

Prompt and adequate antihypertensive therapy is of utmost importance in the management of severe hypertensive disorders in pregnancy [1, 2]. The intravenous anti-hypertensives such as hydralazine (a vasodilator) and labetalol (an alpha and beta receptor blocker), have long been considered the first-line drugs for the management of severe hypertension in pregnancy [1]. However, it is important to note that the availability, affordability, and the presence of skilled personnel to properly administer these drugs are essential for proper utilisation of these drugs [2]. Some of these prerequisites may be lacking in major parts of the low and middle-income countries, thus amplifying the complications and the attendant morbidity and mortality from hypertensive disorders of pregnancy [3].

A recent study has shown that intravenous labetalol, as well as oral immediate-release nifedipine, both anti-hypertensive agents, may be effective in management of severe hypertensive disorders in pregnancy, and hence can be considered first-line drugs [4]. Also, a recent guideline by the American College of Obstetricians and Gynecologists (ACOG) and the Society of Obstetricians and Gynaecologists of Canada (SOGC) included the use of oral nifedipine in the management of hypertensive emergencies in pregnancy especially in situations of difficulty or delay in commencing the intravenous antihypertensives. [5, 6]. Often times, these delays or difficulties may be brought about by obesity or oedema of extremities as may be seen in some women with hypertensive disorders in pregnancy. These scenarios may pose some difficulties in securing intravenous access, especially in emergency situations. The use of effective oral anti-hypertensives will be highly useful in settings where there is imbalance in ‘healthcare personnel to patient ratio’ – a situation commonly seen in low and middle-income countries (LMICs) or where there is very busy referral centers^17,21,23^ with attendant challenge of securing intravenous access for all the patients in need of parenteral medications [7]. It also reduces the need for rigorous monitoring as required for patients on parenteral anti-hypertensive.

Interestingly, there is still not yet a clear consensus on the ideal first-line drug for the management of acute severe hypertension in pregnancy, among the available options [8]. The choice largely depends on cost, availability, race, identified contraindications, and clinician’s experience [9].

The resounding question is: can an oral antihypertensive be as effective as an intravenous anti-hypertensive in the prompt management of severe hypertension in pregnancy? This question has led to some comparative studies between oral antihypertensives (immediate-release nifedipine and labetalol) and intravenous drugs (labetalol and hydralazine) [1, 10–12].

Majority of these comparisons recently were between immediate-release nifedipine and labetalol, as hydralazine is thought to cause a lot of maternal and perinatal complications [13]. However, in LMICs including Nigeria, hydralazine is still being used and has been found to be equally as effective as labetalol, in the management of severe hypertension in pregnancy, with similar maternal and fetal side effects [14]. There is yet to the best of the authors’ knowledge a comparative study from LMICs to ascertain the efficacy and safety of oral immediate-release nifedipine and intravenous hydralazine in the management of severe hypertension in pregnancy. However, a significant gap exists in understanding the efficacy and safety of oral immediate-release nifedipine and an intravenous hydralazine in the control of severe hypertension in pregnancy within the context of randomised control trials. This study therefore was aimed at comparing the efficacy and safety of oral immediate-release nifedipine and an intravenous hydralazine in the control of severe hypertension in pregnancy.

## Methods

This double blind randomised clinical trial was carried out at the labour ward of Nnamdi Azikiwe University Teaching Hospital (NAUTH), Nnewi, Nigeria. The hospital is well equipped with facilities for training both the undergraduate and postgraduate students. The eligible participants were recruited between 20^th^ June 2019 and 20^th^ December, 2019. They included women who presented through the antenatal clinic and labor ward of the NAUTH, Nnewi, and had met the inclusion criteria. All pregnant women at gestational age of ≥ 28 weeks with systolic blood pressure of ≥ 160mmHg, and/or diastolic blood pressure of ≥110mmHg, that persists after 15 minutes of measurement were eligible for the study. The exclusion criteria included eclampsia, signs of imminent eclampsia, severe hypertension complicated by abruptio placentae, or cerebrovascular accident, intrauterine growth restriction, intrauterine fetal death, co-morbidities (asthma, cardiac disease, diabetes mellitus, and other medical disorders in pregnancy), and prior history of adverse drug reaction to immediate-release nifedipine or hydralazine.

The sample size was determined using Charan et al [14] formulae for calculating clinical trials and interventions, for comparison between two groups with quantitative data endpoint. Allowing for 10% attrition, a sample size of 34 participants per group yields 80% power to detect a difference of 10.8 minutes in the needed time to achieve the target blood pressure on the original starting time [15] ranging from 24.0 to 34.8 using a two-sample means test at a two-sided alpha of 0.05, assuming a standard deviation of 15.06.

The eligible women were randomised 1:1 either to oral immediate-release nifedipine and placebo for intravenous hydralazine or placebo for oral immediate-release nifedipine and intravenous hydralazine, using a computer-generated random selection of numbers ((http://www.randomization.com). These numbers were placed into consecutively numbered, opaque (brown) sealed envelopes by an uninvolved third party before the initiation of the study. With the aid of these computer-generated random numbers, eligible candidates were assigned into two groups; A and B which were the intervention group and the control group respectively. Thus, once a patient was deemed eligible and has given informed consent, she was assigned a sequential study number. The third party who was not directly involved in the study was contacted to open the corresponding envelope for the purpose of treatment allocation. Participants randomised to group A were administered “oral immediate-release nifedipine” capsules and also intravenous “normal saline” which served as placebo for hydralazine injection while those randomised to group B were administered an oral cellulose capsule which served as placebo for oral immediate-release nifedipine, and also intravenous hydralazine injection. There was need for “crossover” in some cases where the initial intervention ‘failed’ to achieve the target blood pressure. In order to achieve this and also ensure that the participants and her caregivers (researchers) were unaware of the group they belonged, two brown envelopes labelled ‘Pack A’ and ‘Pack B’ were given to caregivers by the third party. The randomisation group determined the content of each pack. For example; if pack A contained either three capsules each containing active 10mg immediate-release nifedipine and a placebo for hydralazine 20mg (in the form of 20mls of normal saline in a 20ml syringe), then pack B would contain placebo for three capsules of the 10mg immediate-release nifedipine (factory made and looked exactly like the active drugs) and the active hydralazine (Apresoline) 20mg diluted in 20mls of water for injection in a 20mls syringe. The algorithm for administration was as follows: The caregiver was handed over 2 brown envelopes labelled Pack A and Pack B – inside each pack there was a 20mls syringe containing 20mls of either 20mg hydralazine or normal saline placebo, and 3 purple capsules each containing either 10mg immediate-release nifedipine or cellulose placebo. Pack A which was the first pack to be always administered was administered as follows: (i) 5mls of the intravenous drug was given over 5 minutes and also 1 purple capsule was given with a sip of water. (ii) The BP measurement was repeated after 20 minutes. (iii) If within the target blood pressure of systolic 140-150 mmHg, and or diastolic 90-100 mmHg, the research is deemed complete. (iv) However, if BP was still > or = 160/110 mmHg, next step applies which entailed (v) 10mls of the intravenous drug was given over 5 minutes and also 2 purple capsules given with a sip of water. (vi.) The BP measurement was repeated after 20 minutes. (vii) If now within the target blood pressure of systolic 140-150 mmHg, and or diastolic 90-100 mmHg, the research is deemed complete. (viii) If BP is still > or = 160/110 mmHg, next step applies which entailed (ix) Pack B was opened and steps (i) – (vi) were repeated. (x) If the BP remained at or above 160/110mmHg, the research drugs were stopped completely. At this point the researcher is free to use a rescue drug (intravenous labetalol) for the participants. Other principles of managing patients with severe hypertension with or without significant proteinuria were adhered to strictly. Magnesium sulphate was administered when indicated and was noted accordingly.

The researcher and assistants were always on ground till the research was over. They ensured proper adherence to treatment algorithm, and documentation of relevant clinical data including the BP measurements, time of administration of research drugs and achievement of target BP, need for crossover, fetal and maternal side effects, and Apgar scores at delivery.

The BP of all participants were measured using the mercury sphygmomanometer, with the patient seated, and the appropriate-sized cuff placed in the middle one-third of the right arm. The cuff was inflated, with the manometer at the level of the heart, and the inflation was stopped a little above the disappearance of the radial pulsation. The diaphragm of the stethoscope was placed at the cubital fossa, and with gradual release of pressure, the first and fifth Korotkoff was noted as the systolic and diastolic blood pressure respectively. Fetal monitoring was done with either the fetal handheld Doppler or the cardiotocograph as indicated, and fetal Apgar score was noted after delivery.

A forum for communication among the researcher and the research assistants was created using the WhatsApp mobile phone application. As recruitment of participants was being done, a notification of the randomisation sample number utilised was shared, to avoid repetition. Any further clarifications as regards the study were also made on the forum.

The primary outcome measure was the mean time (in minutes) needed to achieve the target BP. The secondary outcome measures included: mean total number of doses of antihypertensives required to achieve the target BP; proportion of participants that required crossover treatment; proportion of participants that required a rescue drug; proportion of participants that had side effects such as headache, nausea and vomiting, hot flushes, palpitations, tachycardia; proportion of fetuses that had fetal heart rate abnormalities; proportion of participants that had complications such as seizures, antepartum haemorrhage, pulmonary oedema, acute kidney injury; proportion of infants in each group that had birth asphyxia (assessed by the fifth minute Apgar score); and the proportion in each group that had vaginal or caesarean section.

The data collected in the proforma was keyed into microsoft excel spreadsheet on daily basis, and the parameters were analyzed using the International Business Machines Corporation — Statistical Package for the Social Sciences (IBM-SPSS) version 24, using the Independent T-test and Chi square test as appropriate. The test of significance was set at a p-value of < 0.05. The study was approved by the Ethics Committee of Nnamdi Azikiwe University Teaching Hospital, Nnewi, Nigeria (identification number: NAUTH/CS/66/VOL.10/216/2017/128). The trial was registered at https://pactr.samrc.ac.za/, PACTR201906662822573. Written informed consent was obtained from study participants.

## Results

**Figure 1:**
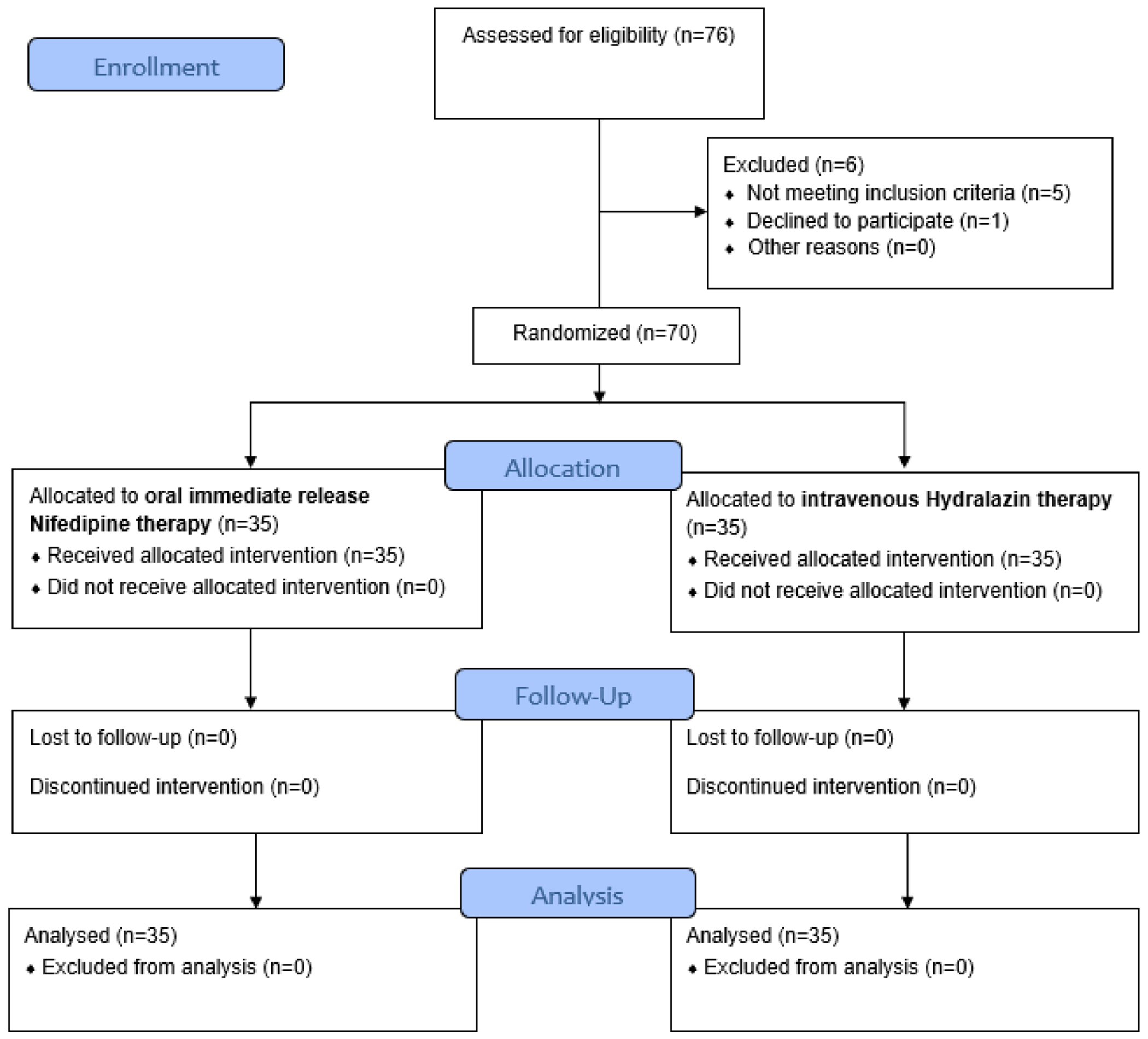
CONSORT flow diagram of the study participants

A total of 79 women were assessed for eligibility and nine were excluded: six due to intrauterine growth restriction (IUGR), two due to eclampsia, and one due to fetal distress at presentation. Thus 70 women were randomised equally into group A (n = 35) and group B (n = 35). Details are as shown in Figure 1. The baseline characteristics of both groups include age, gestational age, parity, educational level, and the initial systolic and diastolic BP are similar (P > 0.05). Details are as shown in Table 1.

The mean time (minutes) taken to reach the target BP for group A and B participants was 48.29 ± 31.95 and 41.20 ± 26.98, respectively. The observed difference was not significant (P = 0.320). Four participants (11.4%) in group B and two in group A (5.7%) could not achieve the target BP in the course of the study (P<0.05). The summary of participants that reached the target BP and those that could not is as stated in Figure 1 and Table 2. The mean doses of antihypertensives used before target BP was reached for participants in group A and B were 1.71 ± 1.02 versus 1.40 ± 0.88 (P>0.05). Thirteen participants (37.1%) in group A had the need to crossover to Group B treatment as against nine participants (25.7%) in group B who needed to crossover to group A treatment. The observed difference was not significant (P = 0.303).

The delivery route (caesarean section versus vaginal delivery) was not different between the two groups (P = 0.643). It was also noted in this study that the major indication for the caesarean sections was severe hypertension alone in 54 (77.1%) patients. However, in the others it was severe hypertension with other associations.

None of the participants in group A or B had headache, tachycardia, palpitations, hot flushes or hypotension. No participant in Group A had nausea and/or vomiting. However, one (2.9%) in group B had nausea and vomiting and another one (2.9%) had only nausea. Fetal heart rate abnormalities were only noted in group B: bradycardia (2.9%, n = 1) and tachycardia (5.7%, n =2) The mean 5^th^ minute Apgar score: group A (9.49 ±1.33) versus group B (9.46 ±1.29) was not significantly different (P>0.05)

There was need to use a rescue drug in two participants (5.7%) in group A versus four participants (11.4%) in group B (P=0.393).

## Discussion

The motivation for this trial was that severe hypertension during pregnancy poses significant risks to both maternal and fetal health, often necessitating prompt and effective treatment. While intravenous hydralazine has been a standard therapeutic option, the administration method can be challenging in resource-limited settings. Oral immediate-release nifedipine offers a potentially safer and more convenient alternative, but its efficacy and safety in this context remain insufficiently evaluated. This study aimed to fill this gap by comparing the efficacy and safety of oral immediate-release nifedipine with intravenous hydralazine in controlling severe hypertension in pregnant women. By establishing the non-inferiority of nifedipine, we aimed to provide evidence supporting its use as a first-line treatment option, particularly in low- and middle-income countries where accessibility to intravenous medications may be limited. The principal findings of the study was that oral immediate-release nifedipine is as effective as intravenous hydralazine in controlling severe hypertension in pregnancy. Both regimen achieved target blood pressure without significant differences in the mean-time taken. Furthermore, the doses required before reaching target blood pressure, the proportion of participants requiring treatment crossover, and those needing rescue antihypertensives (labetalol) were comparable between the two groups (P > 0.05). Importantly, there were no maternal or neonatal side effects reported among participants receiving oral immediate-release nifedipine.

This study showed that the time in minutes required to achieve the target blood pressure was not different in the two groups similar to the findings by Sabir et al [16]. This then implies that both medications are equally effective in achieving the needed target blood pressure in pregnant women with severe hypertension in pregnancy. The same findings was noted by Rezaei et al [15], Firoz et al [17] and Kwawukume and Ghosh [18]. However, these differ from findings of Kaur et al [1], Ehikioya et al [10] and Kausar et al [13] which reported that intravenous hydralazine showed faster achievement of the target blood pressure (BP) and a lower number of doses required compared to intravenous labetalol, and a higher percentage of women in the hydralazine group achieved the target BP with a single dose [1, 10, 13].

However, there were more maternal adverse effects associated with hydralazine, although they were not severe. The average dose of the antihypertensive required to reach the target blood pressure was similar for both groups. This finding however, was different from the studies by Sabir et al [16], where fewer doses of hydralazine were used, and Rezaei et al [15] where fewer doses of nifedipine were used. More studies are therefore encouraged to enable more clarifications for these inconsistencies.

It was noted in this study that there was a need for cross over treatment from either oral immediate-release nifedipine to intravenous hydralazine and vice versa in 31.4% of patients. Furthermore, the study found no statistically significant evidence to associate the cross over treatment to the route of administration of the drug. Interestingly, amongst these same group of patients, the target blood pressure was not reached in 6 (17.1%); four in the intravenous group and two in the oral group. These findings emphasize the idiosyncratic response to drugs by some individuals. Furthermore, it buttresses the need for further study on factors that can affect the pharmacokinetics and pharmacodynamics of the drugs used in this research.

The fetal and maternal side effects are always a source of concern for any drug administered during pregnancy. In this study, there were no documented fetal or maternal side effects for the oral nifedipine group. The absence of maternal side effects, especially the commonly expected headache, as noted in other studies by Rezaei et al [16], Kwawukume and Ghosh [18], and Liu et al [19] was quite surprising; however, the probable explanation could be the short acting nature and duration of intake of the immediate-release nifedipine used in this study. The intravenous hydralazine group, on the other hand had fetal heart rate abnormalities (bradycardia in a patient, and tachycardia in two patients), and maternal side effects which included nausea and vomiting. This was a similar finding noted in Kwawukume and Ghosh study [18]. Also, recent study by Ehikioya et al [10] revealed that there were more maternal adverse effects associated with hydralazine, although they were not severe. This finding calls for a closer monitoring of the feto-maternal vital signs while intravenous hydralazine is being used.

There was no relationship between the route of administration of antihypertensives and the route of delivery of the fetus. This may be explained by the varying factors that determine the route of delivery; majority of which are individualized.

The findings from this study have significant clinical implications for the management of severe hypertension in pregnancy. The study demonstrated that oral immediate-release nifedipine is as effective as intravenous hydralazine in achieving target blood pressure, with a similar mean time to control and comparable dosing requirements. Importantly, the use of oral immediate-release nifedipine was associated with fewer maternal and fetal side effects, making it a safer alternative for pregnant women with severe hypertension. This suggests that oral immediate-release nifedipine could be a preferable first-line treatment in clinical settings, particularly in resource-limited environments where intravenous administration might be challenging.

The absence of serious adverse effects with oral immediate-release nifedipine, compared to the observed side effects with hydralazine, supports its use not only for its efficacy but also for its safety profile. Clinicians might consider switching to or initiating treatment with oral immediate-release nifedipine in similar patient populations to reduce the risk of fetal heart rate abnormalities and maternal discomfort, thus improving overall maternal and fetal outcomes.

This study opens several avenues for future research. First, the results suggest the need for larger multicenter trials to confirm the findings and establish generalisability across different populations and healthcare settings. Additionally, the potential for oral immediate-release nifedipine to be used in a broader range of hypertensive disorders during pregnancy warrants further investigation.

The comparative safety and efficacy profile observed in this study should prompt further research into the pharmacodynamics and long-term outcomes of oral immediate-release nifedipine use in pregnancy. Understanding the mechanisms behind the lower incidence of adverse effects with oral immediate-release nifedipine could guide the development of even more effective and safer antihypertensive therapies for pregnant women. Moreover, exploring patient preferences and adherence to oral versus intravenous treatments could provide valuable insights into optimising hypertension management during pregnancy. These findings also highlight the need for ongoing research into the optimal dosing and administration strategies for antihypertensives in pregnancy, ensuring that maternal and fetal health is prioritised.

The limitation of this study is the small sample size which limited the comparative analysis for some of the secondary outcome measures. The major strength of the study is in its double-blind design which prevented bias and improved the study’ precision and external validity.

In conclusion, the efficacy of oral immediate-release nifedipine and intravenous hydralazine in controlling severe hypertension in pregnancy are similar. These findings indicate that both medications possess a remarkable materno-fetal safety profile, reinforcing the potential of oral immediate-release nifedipine as a viable alternative to intravenous hydralazine in managing severe hypertension during pregnancy. Oral immediate-release nifedipine, is therefore recommended for inclusion in the list of the first-line drugs for control of severe hypertension in pregnancy. This inclusion should be encouraged strongly for the low- and middle-income countries, as it could minimize the complications that often arise from severe hypertension due to delays in reaching centers that can give parenteral medications. Also, the non-invasive nature of oral immediate-release nifedipine makes it an appealing modality especially in resource poor countries. More research is however recommended, preferably on a larger scale, as outcome will likely revolutionize and improve care of patients with severe hypertension in pregnancy especially in low and middle-income countries.

## Figures and Tables

**Table 1:** Baseline demographic and clinical characteristics of participants

Characteristic	Oral Nifedipine (n = 35)	Parenteral Hydralazine (n = 35)	P-Value
**Age (years), mean ± SD**	30.4 ± 4.2	29.8 ± 4.6	0.632
**Gestational age (weeks), mean ± SD**	37.8 ± 1.2	38.0 ± 1.3	0.512
**Parity, n (%)**			
- Nulliparous	12 (34.3)	11 (31.4)	0.811
- Multiparous	23 (65.7)	24 (68.6)	
**Educational Level, n (%)**			0.714
- Primary	6 (17.1)	4 (11.4)	
- Secondary	22 (62.9)	23 (65.7)	
- Tertiary	7 (20.0)	8 (22.9)	
**Initial Systolic BP (mmHg), mean ± SD**	184.6 ± 15.8	185.7 ± 16.7	0.779
**Initial Diastolic BP (mmHg), mean ± SD**	113.3 ± 7.6	114.2 ± 7.4	0.602

**Table 2:** Comparison between oral immediate release nifedipine and intravenous hydralazine

Parameters	Oral immediate release nifedipine (Group A)	Intravenous hydralazine (Group B)	P value
Average time (mins) Taken to reach target B.P (Frequency ± S.D)	48.29±31.95	41.20±26.98	0.320
Participants who did not Reach target B.P	2	4	

Mean dose of anti-hypertensives before target B.P was reached (frequency ± S.D)	1.71 ± 1.02	1.40 ± 0.88	

Patients who received magnesium sulphate Frequency (%)	31	34	

Patients who did not receive magnesium sulphate	4	1	

Maternal side effects of magnesium sulphate:			
Nausea and vomiting	0 (0.0%)	1(2.9%)	
Nausea alone	0 (0.0%)	1(2.9%)	
Fetal side effects:			
Fetal bradycardia	0 (0.0%)	1(2.9%)	
Fetal tachycardia	0 (0.0%)	2(5.7%)	

Mean 5 minute Apgar score	9.49	9.46	

Those who received a rescue drug to achieve target blood pressure (intravenous labetalol)	2	4	0.393

Abbreviations: B,P=Blood pressure; SD=Standard deviation

## Data Availability

Data is provided within the manuscript or supplementary information files.

## References

[1] Kaur T, Kumari K, Rai P, Gupta V, Pandey S, Vineeta, Comparative study of oral nifedipine versus intravenous labetalol for acute hypertension management in pregnancy: Evaluation of feto-maternal outcomes in a hospital-based randomized controlled trial. Int J MCH AIDS. 2024;13. doi: 10.25259/IJMA_660.; PMC11380905 39247143

[2] Yenet A, Nibret G, Tegegne BA. Challenges to the Availability and Affordability of Essential Medicines in African Countries: A Scoping Review. Clinicoecon Outcomes Res. 2023 Jun 13;15:443–458. doi: 10.2147/CEOR.S413546. 37332489 PMC10276598

[3] Gudeta TA, Regassa TM. Pregnancy Induced Hypertension and Associated Factors among Women Attending Delivery Service at Mizan-Tepi University Teaching Hospital, Tepi General Hospital and Gebretsadik Shawo Hospital, Southwest, Ethiopia. Ethiop J Health Sci. 2019 Jan;29(1):831–840. doi: 10.4314/ejhs.v29i1.4. 30700950 PMC6341446

[4] S D, Novri DA, Hamidy Y, Savira M. Effectiveness of nifedipine, labetalol, and hydralazine as emergency antihypertension in severe preeclampsia: a randomized control trial. F1000Res. 2023 Apr 27;11:1287. doi: 10.12688/f1000research.125944.2. 37273965 PMC10238817

[5] Braunthal S, Brateanu A. Hypertension in pregnancy: Pathophysiology and treatment. SAGE Open Med. 2019 Apr 10;7:2050312119843700. doi: 10.1177/2050312119843700. 31007914 PMC6458675

[6] Magee LA, Smith GN, Bloch C, Côté AM, Jain V, Nerenberg K, Guideline No. 426: Hypertensive Disorders of Pregnancy: Diagnosis, Prediction, Prevention, and Management. J Obstet Gynaecol Can. 2022 May;44(5):547–571.e1. doi: 10.1016/j.jogc.2022.03.002. 35577426

[7] Nyame S, Boateng D, Heeres P, Gyamfi J, Gafane-Matemane LF, Amoah J, Community-Based Strategies to Improve Health-Related Outcomes in People Living With Hypertension in Low- and Middle-Income Countries: A Systematic Review and Meta-Analysis. Glob Heart. 2024 Jun 12;19(1):51. doi: 10.5334/gh.1329. 38883258 PMC11177843

[8] Alavifard S, Chase R, Janoudi G, Chaumont A, Lanes A, Walker M, Gaudet L. First-line antihypertensive treatment for severe hypertension in pregnancy: A systematic review and network meta-analysis. Pregnancy Hypertens. 2019 Oct;18:179–187. doi: 10.1016/j.preghy.2019.09.019. 31678759

[9] Duley L, Meher S, Jones L. Drugs for treatment of very high blood pressure during pregnancy. Cochrane Database Syst Rev. 2013 Jul 31;2013(7):CD001449. doi: 10.1002/14651858.CD001449.pub3. 23900968 PMC7073408

[10] Ehikioya E, Okobi OE, Beeko MAE, Abanga R, Abah NNI, Briggs L, Comparing Intravenous Labetalol and Intravenous Hydralazine for Managing Severe Gestational Hypertension. Cureus. 2023 Jul 23;15(7):e42332. doi: 10.7759/cureus.42332. 37614273 PMC10443893

[11] Patel P, Koli D, Maitra N, Sheth T, Vaishnav P. Comparison of Efficacy and Safety of Intravenous Labetalol Versus Hydralazine for Management of Severe Hypertension in Pregnancy. J Obstet Gynaecol India. 2018 Oct;68(5):376–381. doi: 10.1007/s13224-017-1053-9. 30224842 PMC6133790

[12] Easterling T, Mundle S, Bracken H, Parvekar S, Mool S, Magee LA, Oral antihypertensive regimens (nifedipine retard, labetalol, and methyldopa) for management of severe hypertension in pregnancy: an open-label, randomised controlled trial. Lancet. 2019 Sep 21;394(10203):1011–1021. doi: 10.1016/S0140-6736(19)31282-6. 31378394 PMC6857437

[13] Kausar M, Husain S, Hussain R. Comparison of efficacy of intravenous labetalol and intravenous hydralazine for management of pre-eclampsia in pregnant women. Afri Health Sci. 2023;23(1):320–5. 10.4314/ahs.v23i1.34. PMC10398458 37545898

[14] Charan J, Biswas T. How to calculate sample size for different study designs in medical research? Indian journal of psychological medicine. 2013;35(2):121. 24049221 10.4103/0253-7176.116232 PMC3775042

[15] Rezaei Z, Sharbaf FR, Pourmojieb M, Youefzadeh-Fard Y, Motevalian M, Khazaeipour Z, Comparison of the efficacy of nifedipine and hydralazine in hypertensive crisis in pregnancy. Acta Medica Iranica. 2011;49(11):701. 22131238

[16] Sabir S, Yasmin S, Abbas G. Comparison of oral nifedipine with intravenous hydralazine for acute hypertensive emergencies of pregnancy. Journal of Postgraduate Medical Institute (Peshawar-Pakistan). 2016;30(4).

[17] Firoz T, Magee L, MacDonell K, Payne B, Gordon R, Vidler M, Oral antihypertensive therapy for severe hypertension in pregnancy and postpartum: a systematic review. BJOG: An International Journal of Obstetrics&Gynaecology. 2014;121(10):1210–8. 24832366 10.1111/1471-0528.12737 PMC4282072

[18] Kwawukume E, Ghosh T. Oral nifedipine therapy in the management of severe preeclampsia. International Journal of Gynecology & Obstetrics. 1995;49(3): 265–9. doi: 10.1016/0020-7292(95)02372-j 9764864

[19] Liu Q, Yu Y, Gong S, Huang L. Clinical efficacy and perinatal outcome of nifedipine for severe preeclampsia: meta-analysis. Zhonghua fu chan ke za zhi. 2012;47(8):592–7. 23141179

